# Correlating Estrogen Levels and Cognitive Functions in Regularly Menstruating Females of Reproductive Age Group and Post Menopausal Women of North India 

**Published:** 2015-06

**Authors:** Deepti Khattar, Candy Sodhi, John Parmod, Abhilasha Dutta

**Affiliations:** 1 Department of Physiology, University College of Medical Sciences, Delhi, India; 2 Department of Physiology, Christian Medical College, Ldh, Punjab, India; 3 Department of Physiology, Index Medical College, Indore, M.P., India

**Keywords:** cognitive function, post menopausal women, Mini-Mental State Examination and serum estrogen

## Abstract

**Objective:** To correlate serum estrogen levels with cognitive functions calculated objectively as per Mini Mental State Examination (MMSE) in the females in reproductive age group and those attaining menopause.

**Materials and methods:** This study was conducted in Christian Medical College and Hospital, Ludhiana, Punjab, India. 150 subjects (100 postmenopausal females and 50 regularly menstruating females of the reproductive age group) were included. The cognitive functions of all the females and serum estrogen levels (i.e Estradiol E2) were assessed.

**Results:** The E2 levels in normal menstruating females were found to be higher (mean = 188.062 pg/ml) as compared to the menopausal females and the difference in E2 levels was found to be significant (p < 0.001). However, the difference in serum estrogen levels of subjects in the two menopausal groups was insignificant. MMSE, showed that scores of normal menstruating females were higher (mean score = 29.92) as compared to post menopausal females for 1-5 years (mean score = 26.72) and post menopausal females for last 6-10 years (mean score = 26.30).

**Conclusion:** We observed that the cognition functions declined in post menopausal women, whereas the scores were higher in the women of reproductive age group, meaning thereby, that it is the serum estrogen level that is bringing about this difference. Another finding was that the decline in cognition following menopause was not progressive. Therefore, this correlation would open up the gates for the use of estrogen therapy for various neuropsychological disorders pertaining to cognition in the postmenopausal females.

## Introduction

The term cognition implies the thinking processes of the brain, using both the sensory input to the brain and the information already stored in the memory. Most of our motor actions occur as a consequence of thoughts generated in the mind, a process called cognitive control of motor activity (1).

Physiologically, it is an evoked response to a previously stored experience of a set of stimuli. It is a complex phenomenon which relies on neurotransmitter processes like serotonin, dopamine, GABA to coordinate the signals that are sent between the different areas of the brain like the hippocampus and prefrontal cortex, which serve the episodic and working memory (2).

Estrogen is believed to have a direct influence on the brain function pertaining to cognition. Even in healthy older women, brain volume begins to decline as estrogen levels fall in the perimenopausal period. This atrophy occurs particularly in the hippocampus and parietal lobe areas primarily associated with memory (3). Loss of cognitive function also can be related to endogenous estrogen deficiency. Another effect of menopause and loss of estrogen on the brain is a slowdown in the speed of brain processing (3).

Several studies have been conducted to evaluate the effect of estrogen replacement therapy on cognitive functions and related physical and physiological factors. Baltimore Longitudinal Study of Ageing (BLSA) says that ERT offers a selective benefit to specific memory processes (4). Another study named Prospective 3C Study, (2009) showed that current HT was associated with better performance in certain cognitive domains but these associations are dependent on the duration and type of treatment used (5). 

However, all studies do not show a clear benefit or association of estrogen levels and cognitive functions. The results of the Women’s Health Initiative Memory Study (WHIMS) suggested that women taking either conjugated equine estrogens alone or combined with medroxyprogesterone acetate had higher risk of dementia than those taking placebo (6). 

Interestingly, The Rotterdam Study (7) of 3600 postmenopausal women defies the claim of beneficial effects of estrogen on cognition. In fact it concludes that a longer exposure to endogenous estrogen may selectively increase the risk of incident dementia in postmenopausal women.

There are also studies like the Study of Women's Health Across the Nation (SWAN) (2007) which found no association between phase of the menopausal transition (or estradiol levels) and several tests of cognitive performance (8). Also, in the Melbourne Women's Midlife Health Project memory was similar regardless of menopausal status, number of years post menopause, current or prior use of estrogen, or duration of hormone treatment, and was also unrelated to blood estradiol levels (9).

Several tools and tests, (for e.g., Bender-Gestalt Test (10), Wechsler Memory Test (11), Halstead-Reitan Categories Test (12), Trail Making Tests (13) have been devised and standardized by physiologists and cognitive scientists to assess cognitive functions at different age groups. 

The Mini-Mental State Examination (MMSE) (14) or Folstein test as specified in the Harrison’s Principles of Internal Medicine is a standard test for testing and grading cognitive status and is widely used in many studies as a screening tool.

Efforts must, therefore, be made to assess the cognitive functions of post menopausal women in different chronological ages of menopause in order to correlate the levels of circulating endogenous estrogens and cognitive functions as assessed by standard tools of cognitive function testing.

To correlate physiological status of cognitive functions calculated objectively as per Mini Mental State Examination with serum estrogen levels in premenopausal females and those who had attained menopause for a period extending over 1-10 years.

## Materials and methods

This study was conducted in the Department of Physiology in collaboration with the Department of Biochemistry and Psychiatry of Christian Medical College and Hospital, Ludhiana. The study was initiated after receiving formal approval by Research and Ethics Committee of CMC Ludhiana, Punjab, India. All subjects gave their formal consent for participation as well as for one time estimation of serum estrogen levels.

One hundred and fifty female subjects > 18 years (for control group) and < 60 years (for all three groups) were randomly chosen from the general community which also included volunteers from the staff of the hospital and college, and the general population. 

The following subjects (both control as well as of the study group) were excluded from this study:

     1.History of recent or current Estrogen or hormonal replacement therapy.

     2.Known hypertensive, diabetic, renal disease, endocrine disorders, overt nutritional deficiency states, anemia, liver disease or those having history of psychiatric illness.

     3.Women having estrogen secreting ovarian tumors, for e.g., granulosa and theca cell tumors, tumors of the adrenal gland.

     4.History of chronic smoking or regular alcohol intake

     5.History of treatment with pioglitazone, which induces estrogen metabolism by enhancing the activity of the cytochrome P4503A4 enzyme.

     6.Women who are on drugs that can increase estrogen levels, for e.g., steroid medications, phenothiazines, ampicillin, estrogen-containing drugs, tetracycline, digitalis, gonadotropins and cimetidine.

     7.Women who are on drugs that can decrease estrogen levels by enhancing its metabolism by the liver, e.g., rifampicin, barbiturates, carbamazepine, griseofulvin, phenytoin, primidone and warfarin.

     8.Abnormal estrogen levels in subjects of control group.

     9.Known history of neurological disorders including CVA, space occupying lesions, epilepsy and developmental anomalies.

     10.Women, who are physically unable to hear, read or understand written or explained instructions properly, or may have a motor deficit that affects writing and drawing skills. Table 1.

The serum estrogen levels were estimated in the Department of Biochemistry, Christian Medical College and Hospital, Ludhiana. 

Serum estrogen levels vary during the different phases of the menstrual cycle and during the different times of the day, so, to maintain uniformity in the control group, the blood samples were drawn during mid-cycle days (as per the individual’s menstrual history). For each subject 5 ml of blood specimen was drawn between 8am to 9am. Serum estrogen levels were estimated using fully automated chemiluminescence Elecsys 2010 analyzer with kits obtained from Rosche Diagnostics. The cognitive functions of all the females were assessed objectively. MMSE included simple questions and problems as per the details given in the Table 2. Any score greater than or equal to 25 points (out of 30) is effectively normal (intact). Below this, scores can indicate mild (21-24 points), moderate (10-20 points) or severe (</ = 9 points) cognitive decline. Comparative analysis of the cognitive functions and serum estrogen levels were done in the two groups of women of normal menstruating and post menopausal females. Assessment of the correlation between physiological decline in estrogen levels and cognitive functions of postmenopausal women was statistically analyzed using ANOVA and other relevant tests of statistical significance.

**Table 1:** Division of subjects into three study groups

Study Group	Subjects
**I**	**50 subjects**
** Subjects who have been normally and regularly menstruating for at least 1 year and not on regular oral contraceptives or hormonal therapy.**	
**II**	**50 subjects**
** Menopausal women (taken as complete cessation of menstruation for at least 1 year/12 cycles) who had attained menopause for a period extending over 1 – 5 years at the time of inclusion into the study.**	
**III**	**50 subjects**
** Menopausal women (taken as complete cessation of menstruation for at least 1 year/12 cycles) who had attained menopause for a period extending over 6 – 10 years at the time of inclusion into the study.**	

**Table 2:** Components of Mini – Mental State Examination

Category	Possible points	Description
**Orientation to time**	**5**	**From broadest to most narrow. Orientation to time has been correlated with future decline.**
**Orientation to place**	**5**	**From broadest to most narrow. This is sometimes narrowed down to streets, and sometimes to floor.**
**Registration**	**3**	**Repeating named prompts.**
**Attention and calculation**	**5**	**Serial sevens or spelling “world” backwards. It has been suggested that serial sevens may be more appropriate in a population where English is not the first language.**
**Recall**	**3**	**Registration recall.**
**Language**	**2**	**Name a pencil and a watch or brand of an object**
**Repetition**	**1**	**Speaking back a phrase.**
**Complex commands**	**6**	**Varies. Can involve drawing figure/s as shown to the subject which are easy to recall and copy e.g. interlocking pentagons**

## Results

The present study was aimed at correlating serum estrogen levels with physiological status of cognitive functions in normal menstruating and postmenopausal females.

The following observations were made:

The age of females in group I ranged between 19-50 years and that in groups II and III between 40-59 years.The education level of all the subjects participating in the study was assessed and it was observed that the education level of the subjects did not influence their score on the MMSE.

Determination of serum estradiol (E2) levels of all the females enrolled in the study showed that, E2 levels were higher in group I females (56.8-596 pg/ml) than group II (5-122.8 pg/ml) and group III (5-131.8 pg/ml).It was observed that E2 levels decline following menopause, alongside it was also observed that this decline does not show a linear relationship with increasing number of years of menopause. 

Mini-Mental State Examination (MMSE), the test for cognitive functions, of all the subjects showed that MMSE score of group I females was higher (mean score = 29.92) as compared to group II (mean score = 26.72) and group III (mean score = 26.30**). **The difference in MMSE scores between the normal menstruating and postmenopausal females was found to be statistically significant (p < 0.001), but the difference in MMSE scores amongst the two groups of postmenopausal females (i.e. group II & III) was not statistically significant. Therefore, we observed that cognitive functions decline following menopause, although the fall was not found to be progressive.The MMSE score followed the same pattern, meaning thereby that cognitive functions (assessed by the MMSE) show a decline as the serum estrogen levels fall following menopause, but, the decline in serum estrogen levels is not progressive as the duration of menopause progresses.

## Discussion

Cognitive decline is deterioration in cognitive function. It is characterized by increasing difficulties with memory (new learning), speed of information processing, language and other cognitive functions. Postmenopausal women with higher levels of endogenous estradiol also have better semantic memory than do those deficient in the estrogen. 

The present study size corresponds to that reported in the COGENT Trial (15). However there are larger studies reported in the literature as having varied number of subjects, 425 by Yaffe et al (16), 837 in the Kame Project (17), 1889 in the Cache County Study (18), and 800 in the recent ,Rancho Bernardo Study(19).

The present study also considered assessing the education level of all the subjects participating in the study. Our study found that the education level of the subjects did not influence their score on the MMSE. In contrast to this, Rosselli et al, observed that education had a significant effect on the MMSE scores of the subjects included in their study (20). 

Group I was taken as the “control” group and Groups II and III were taken as the “study” groups. Cognitive functions of all the females were tested using MMSE (Mini-Mental State Examination) which is a test for global cognitive functions. In our study, it was found that MMSE score of group I females was higher (mean score = 29.92) as compared to group II (mean score = 26.72) and group III (mean score = 26.30). There was a significant difference in the MMSE scores of group I when compared with group II (p < 0.001) and also when compared with group III (p < 0.001), although the difference in the scores amongst groups II and III was insignificant. 

Also serum estrogen levels (Estradiol E2) of all the females enrolled in our study were assessed. The E2 in females of the control group was found to be higher (mean = 188.062 pg/ml) as compared to that in the study groups. This difference in E2 levels between the control group and either of the study groups was found to be significant (p < 0.001). However, the difference in serum estrogen levels of females in the two study groups was insignificant. 

Therefore, from the present study we observed that serum estrogen levels of females in the fertile age group were higher. After the females attained menopause (i.e. in females belonging to the menopausal age group), these levels declined. However, this decline in serum estrogen levels was not found to be progressive with increasing number of years of menopause. 

Some studies similar to our study have been found in literature. A study by Yaffe et al (16) observed that cognitive impairment (a decrease of 3 points or more in MMSE score) occurred in 16% of women in the low titer of endogenous estradiol. Another large cross-sectional Dutch Study (21) found that women in the highest quintile of either estradiol or estrone were 40% less likely to be cognitively impaired (MMSE < 27) compared to women in the lowest quintile.

On the other hand, we have studies that do not agree with the view that endogenous estrogen is protective for cognition. A recent study, The Rancho Bernardo Study, observed that women in the highest textiles of neither estrone nor bioavailable estradiol were associated with any change in MMSE scores (19). 

MMSE is a test of global cognitive functioning and includes the testing of various aspects of cognition like - orientation, immediate memory, attention/concentration, delayed recall and language. In our study all these components of cognition were tested in all the women participating in the study and the scores of each component were compared amongst the three groups, to determine precisely, the area of cognition that was affected with declining estradiol levels in postmenopausal women.

## Conclusion

Hence, from the present study it can be concluded that the menopausal age for North Indian subjects is variable and is associated with significant decline in serum estrogen levels once the menopause sets in. The study also highlights the fact that there is significant decrease in the cognitive function in the post menopausal period. However, not much of change was seen with the increasing menopausal years up to 10 years, once the menopause has been established. 

There are plausible biological and neurophysiologic mechanisms that might account for a beneficial effect of estrogen therapy on cognition, a reduced risk for developing dementia, or improvement in the severity of dementia. Therefore, this correlation would open up the gates for the use of estrogen therapy for various neuropsychological disorders pertaining to cognition in the postmenopausal females.
